# The Importance of Weakly Co-Evolving Residue Networks in Proteins is Revealed by Visual Analytics

**DOI:** 10.3389/fbinf.2022.836526

**Published:** 2022-04-05

**Authors:** Sidharth Mohan, Hatice Gulcin Ozer, William C. Ray

**Affiliations:** ^1^ Interdisciplinary Graduate Program in Biophysics, The Ohio State University, Columbus, OH, United States; ^2^ Lilly Research Laboratories, Eli Lilly and Company, Indianapolis, IN, United States; ^3^ The Battelle Center for Mathematical Medicine, The Research Institute at Nationwide Children’s Hospital, Columbus, OH, United States

**Keywords:** proteins, correlations, evolution, structure, contact, visualization, analytics

## Abstract

Small changes in a protein’s core packing produce changes in function, and even small changes in function bias species fitness and survival. Therefore individually deleterious mutations should be evolutionarily coupled with compensating mutations that recover fitness. Co-evolving pairs of mutations should be littered across evolutionary history. Despite longstanding intuition, the results of co-evolution analyses have largely disappointed expectations. Regardless of the statistics applied, only a small majority of the most strongly co-evolving residues are typically found to be in contact, and much of the “meaning” of observed co-evolution has been opaque. In a medium-sized protein of 300 amino acids, there are almost 20 million potentially-important interdependencies. It is impossible to understand this data in textual format without extreme summarization or truncation. And, due to summarization and truncation, it is impossible to identify most patterns in the data. We developed a visualization approach that eschews the common “look at a long list of statistics” approach and instead enables the user to literally look at all of the co-evolution statistics simultaneously. Users of our tool reported visually obvious “clouds” of co-evolution statistics forming distinct patterns in the data, and analysis demonstrated that these clouds had structural relevance. To determine whether this phenomenon generalized, we repeated this experiment in three proteins we had not previously studied. The results provide evidence about how structural constrains have impacted co-evolution, why previous “examine the most frequently co-evolving residues” approaches have had limited success, and additionally shed light on the biophysical importance of different types of co-evolution.

## Introduction

Proteins are the molecular machines responsible for carrying out practically every chemical reaction in a living organism. Their complexity is immense and yet, the standard method for describing them is as a simple catalog of the parts they contain. The 3-dimensional structures of only a relative handful of proteins have been determined, and so for them, somewhat more detailed information is available. However, for the vast majority, researchers are left trying to study the inner workings of molecular machines every bit as complex as a car engine, using nothing more than a list of their parts, with no assembly diagram.

Fortuitously, even without an assembly diagram, evolution has frequently left important clues about function scattered throughout the parts catalog. Since the basic biological needs of life are similar across the entirety of the phylogenetic tree, evolution has tended to reuse the same proteins, with minor modifications to carry out the same functions in many different organisms. For example, the molecular machine responsible for breaking down complex sugars into simple sugars is similar across all species, from bacteria to humans. The same idea extends to molecular machinery for a variety of other biochemical processes. While random mutations have introduced changes to the machinery in each variety of organism across evolutionary history, the shape and functioning of the machines have remained largely similar (Orengo et al., 1997; Altschul et al., 1997).

It is these collections of molecular machines with largely similar shape and function, but with changed parts lists, that provide protein researchers with some of the most useful insights into how proteins work (Steipe et al., 1994). By statistically analyzing what parts are allowed to change, what parts can be substituted, and when various parts must be swapped for differing assemblies simultaneously or not at all, protein scientists can infer which pieces of the machines work together and which pieces are independent. Because proteins are 3-dimensional machines and the parts must fit together, pieces that must work together can imply structural relationships, and so ideas about working assemblies can be constructed even from just lists of parts (Perrier et al., 1998). Identifying co-evolving parts of proteins *via* statistical means extracts information not just from a specific protein in a specific evolutionary context, but also from all the similar proteins across all the known organisms, presenting an abundance of data (Durbin et al., 1998; Krogh, 1998; Ray et al., 2009).

The remaining challenge is that this abundance of data that can be inferred by statistically comparing the parts lists, or protein sequences as they are called, is staggering. A typical protein may have 300 amino-acid residue building blocks from which it is composed. Each of these building blocks can have one of 20 shapes (or be absent), and they can fold up and interact in a nearly uncountable variety of ways. As a result, even if only pairwise combinations of residues are considered as possible sub-assemblies, when co-evolution is considered, the collection of available data grows from a 300-item parts list to a nearly 20-million-item long list of potentially cooperating combinations of parts. Of course, the amino-acid parts of proteins interact in groups much larger than pairs, so the real size of the data is orders of magnitude larger.

For years, researchers have been trying to define better statistics for co-evolution, and one of the tests commonly applied, is whether the predicted co-evolving residue pairs are in proximity. It is a reasonable intuition that parts of an assembly that affect each other are probably near each other. Therefore, to determine whether one’s approach for identifying co-evolution is valid, checking the proximity of things that most confidently co-evolve, seems like a reasonable first-pass test (Halperin et al., 2006).

Unfortunately, repeated attempts to predict residue proximity from different estimates of co-evolution have had only limited success. The most successful have either been restricted to specific proteins, placed cumbersome restrictions on the sequences analyzed, or applied complex models from which biological understanding is hard to extract (Halperin et al., 2006; Gomes et al., 2012; Ovchinnikov et al., 2014; Jia et al., 2020). Machine Learning has demonstrated that co-evolution data, in its entirety, does contain information about structure (Schaarschmidt et al., 2018; Salmanian et al., 2020; Li et al., 2021). In particular AlphaFold and AlphaFold2 have shown that deep learning that incorporates covariation in homologous sequences can significantly improve the prediction of tertiary structure (Senior et al., 2020; Jumper et al., 2021). However, these powerful Machine Learning tools for structure prediction provide minimal insight into where the information that they employ is hiding. And, current attempts to unravel this mystery are focusing on tightly-constrained, tractable subsets of the problem such as nonadditive triangular couplings (Werner et al., 2021). Overall, co-evolution has empowered useful predictions, but a broad view of what information lives in co-evolution data, and where it lives in that data, has remained disappointingly elusive.

The core of the problem with inferring a relationship between co-evolution data and structural information, is that there is simply too much data. The fingerprints implicated in specific contexts, such as those of the structural necessities of neighboring residues fitting each other, exist amongst millions of other related fingerprints, and extracting useful information from noisy protein sequence data is a daunting task (Lee and Kim, 2009). While sophisticated approaches such as deep learning demonstrate that the information is present, simple techniques for reducing the data down to interesting subjects for analysis, such as picking the statistically most strongly co-evolving pairs of residues and discarding the rest, have not been successful (Halperin et al., 2006; Gomes et al., 2012; Li et al., 2021).

**FIGURE 1:**
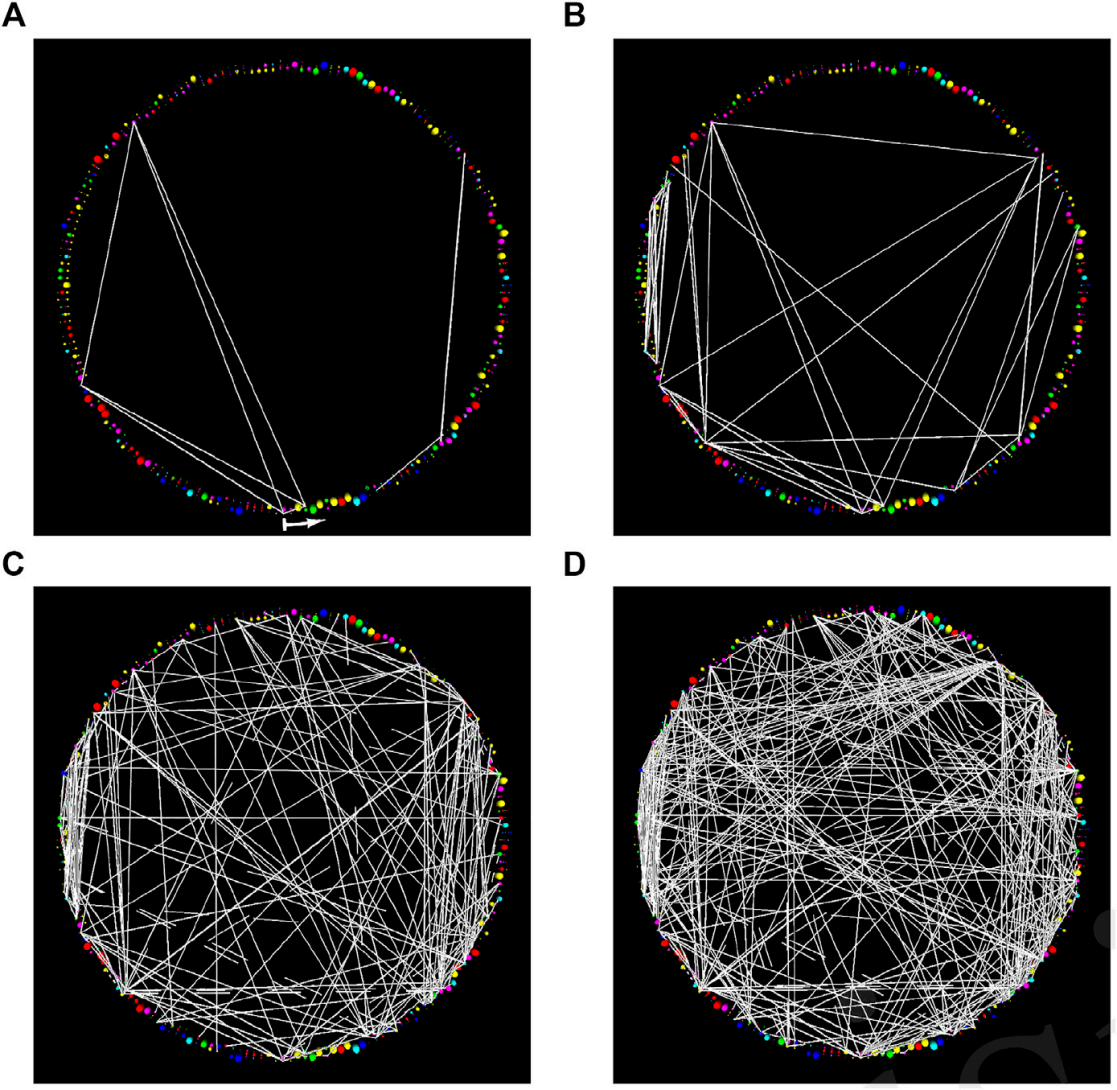
StickWRLD presents the user with an interactive interface to a (pseudo) radial-layout node-link diagram of residue co-evolution statistics. The family (residue identity) position-specific scoring matrix (PSSM) is arranged sequentially around the periphery of a cylinder and edges connecting co-evolving residues are drawn between their corresponding PSSM positions. These diagrams can contain as much or as little of the complete set of co-evolution statistics as the user desires, and can be rapidly “dithered” around any given set of parameters to see how small changes in parameter choice change the displayed subset of residue-pair statistics. In this figure, diagrams show StickWRLD visualizations for the correlated mutations within adenylate kinase, extracted solely from the Pfam (Punta et al., 2012) sequence alignments. They are arranged in order of decreasing statistical significance. The visually salient “clouds” or clusters of edges visible in **(D)** are indicative of structural contacts, despite the fact that at *p* ≤ 0.1 they are well within the noise floor. *T*_*r*_ > = 0.15 for all images. The start of the arrow in subfigure **(A)** indicates the N-terminal of the protein and it points in the direction of increasing sequence coordinates. **(A)** The most significant correlations, with *p* ≤ 0.005, have no obvious visual pattern. **(B)** As the significance is weakened, here *p* ≤ 0.010, more correlations appear. **(C)** When correlations with *p* as poor as 0.050 are shown, distinct patterns begin to appear in the cloud of minimal-significance correlations. **(D)** Even when the significance is only *p* ≤ 0.100, the cloud of weak correlations remains visually focused around certain areas in the diagram.

Applying a simple visualization approach (Ray, 2004)^1^ to “all of” of the co-evolution data for a protein however—even with quite limited family data available—demonstrates an interesting phenomenon. When co-evolution data is filtered using a weak statistical significance threshold, visually salient features appear, and the residues implicated by these features appear to have structural relationships: they are almost ubiquitously closer than expected for residues with similar sequential separation in a protein. As the threshold of statistical significance is made more stringent however, the visually salient groups disappear. Moreover, the few residue pairs remaining at the most stringent thresholds often display no structural relationship (Ozer, 2008). An example of this phenomenon in the adenylate kinase (ADK) family (Berry and Phillips Jr., 1998) is shown in Figure 1.

Put another way, those patterns of co-evolution that appear to be the most necessary—those that would appear at the top of a list sorted by significance—don’t appear to be related to structure (Ozer and Ray, 2006), while those scattered further down in the list—their concerted relationships only observable by literally looking at the patterns they make—seem to be implicated in protein structure.

To determine whether this result was an anomaly, or whether visually-salient patterns of weak co-evolution actually provided signals of structural information, we tested similarly visually-salient patterns of co-evolution in other proteins. Our results suggest that the community intuition—co-evolution contains information about structure—and many of the simple approaches for detecting that co-evolution, have been right all along, but that counter-intuitively, the information resides in patterns of more-weakly co-evolving residues rather than the residue pairs that display the strongest co-evolution signal.

## Methods

### Experimental Design in Brief

In our study we applied our visualization tool StickWRLD (Ray, 2004; Ozer and Ray, 2006, 2007; Ray et al., 2014) to identify co-evolutionary changes in protein families that fell into visually interesting groups. This work was performed by a user with StickWRLD experience, using protein families with which he had no prior experience. Inter-residue distances between the “interesting” residue pairs in a protein data bank (PDB) Berman et al. (2000) structure from each family were determined. To confirm the visual salience of the residue-residue correlations selected by this user, static images from his StickWRLD sessions were shown to a group of 32 university students and their selections of “interesting” groups recorded. To further confirm that visual salience was a property of the protein families and sequences rather than random noise or an artifact, the same 32 students were asked to make selections in randomized StickWRLD images as well. Finally, to confirm that the bias towards unexpectedly-short inter-residue distances was a property of the selected residue pairs rather than a general property of residues with similar sequential separation along the sequences, the observed inter-residue distances were compared to the entire list of inter-residue distances for residues with each pair’s separation along the protein sequence.

### StickWRLD

StickWRLD was originally conceived as a hypothesis-generating tool for exploring patterns of co-evolution in RNA and protein families, with the intent of enabling better sequence-homology searches (Ray, 2004; Ozer and Ray, 2006). As a result it provides many more analytical and presentation capabilities than were used in this study. In general terms, StickWRLD visualizes the residue distribution found in each position of a sequence alignment and the joint distribution of residue pairs between positions of the alignment. It presents a view of the amino-acid utilization of the a family of proteins with similar function. At each sequential position in order of assembly, it shows the distribution of parts observed to be used. It projects into this view, information regarding when pairs of positions are interdependent, as evidenced by simultaneous changes across evolutionary history.

As used here, StickWRLD presents the user with an interactive interface to what is essentially a radial layout of the protein sequence positions, and projects weighted node-links into this layout to convey information regarding the frequency with which different pairs of amino acids apparently co-evolve.

The display differs from a strictly 2D/planar radial presentation such as Circos (Krzywinski et al., 2009) by using the third dimension to enable each sequential radial position to encode all 20 possible amino acids, and the co-evolution node-links connect the individual amino acid identities rather than just the columns. This departure from a planar 2D presentation enables the interface to display (potentially) every possible node-link between every possible pair of amino acid identities across the entire protein family, and for the user to explore this large volume of data in its entirety.

The individual sub-nodes for each individual amino acid in a sequence position are scaled to the population density of that amino acid in that position of the protein family. The node-links between amino-acid identities at different positions are scaled to the unexpected (Ray, 2004) population of sequences sharing those two amino acids. We call the unexpected population *T*_*r*_, or the Total residual, as it is the difference between the observed population sharing the two amino acids, and the expected population that would share them if the amino acids selected by the protein family at those positions were independent.

StickWRLD enables the user to filter the displayed subset of node-links based on *T*_*r*_, statistical significance, and a number of other parameters. The filter parameters can be changed easily using increment-decrement buttons or simple sliders, enabling the user to rapidly “dither” between different filter settings to see how the displayed information changes.

### Protein Family Selection

Having noted a surprising consistency in the visual salience of the “interesting clouds” that we previously observed in ADK, and with evidence that the co-evolving residues in three such patterns were all closer than expected, we undertook to determine if patterns of similar visual salience in other proteins were also signatures of structural proximity.

A student familiar with both protein biophysics and StickWRLD selected three protein families from Pfam (Punta et al., 2012) that met the following criteria: Proteins in the families were less than 500 amino acids in length; At least 50 members in the Pfam seed-sequence list for the family; The seed sequences had no large ( >25% of the sequences and >25% of the family length) gaps; The family displayed similar “interesting clouds” at similar levels of our *T*_*r*_ threshold and statistical significance to the patterns we had previously identified in ADK (*T*_*r*_ ≥ 0.1, *p* ≤ 0.1); At least one protein structure needed to be available in the Protein Data Bank.

Without consulting the content of the protein structure files, the student selected the Chain G of the gelsolin family (PF00626) (Silacci et al., 2004), the P-II family (PF00543) (Cheah et al., 1994), and the X8 domain family (PF07983) (Barral et al., 2004), with corresponding PDB entries 1NM1, 1HWU, and 2JON for further analysis.

The student loaded the seed sequence alignment for each selected family into StickWRLD and explored the interdependencies until “interesting clouds” appeared, then recorded the residue pairs involved in these clouds (Supplementary Material). A static view of the StickWRLD display with these parameters was saved.

The columnar order of each protein family was then randomized, the resulting alignments loaded into StickWRLD and static images again saved with the same parameters as before. It is important to note that this maintains exactly the same set (number and size) of nodes and node-links as appear in the original images, but randomizes their placement in the radial layout.

The PDB structures were then analyzed to determine the expected inter-residue distance for each possible sequential-separation in that protein structure, and to determine the inter-residue distances of any residue pairs that corresponded to those pairs selected from the StickWRLD exploration of the sequence family.

### Confirmation of the Visual Salience of “Interesting Clouds”

The static images of StickWRLD displays for each of the protein families, as well as their randomized counterparts were presented as printed copies to 32 undergraduate and graduate-level computer-science students with no experience with protein science. The students were instructed to select “visually interesting regions” in each display by circling them on the page, and their answers were recorded.

## Results

### Weak Patterns of Co-evolution Consistently Implicate Structure

**FIGURE 2:**
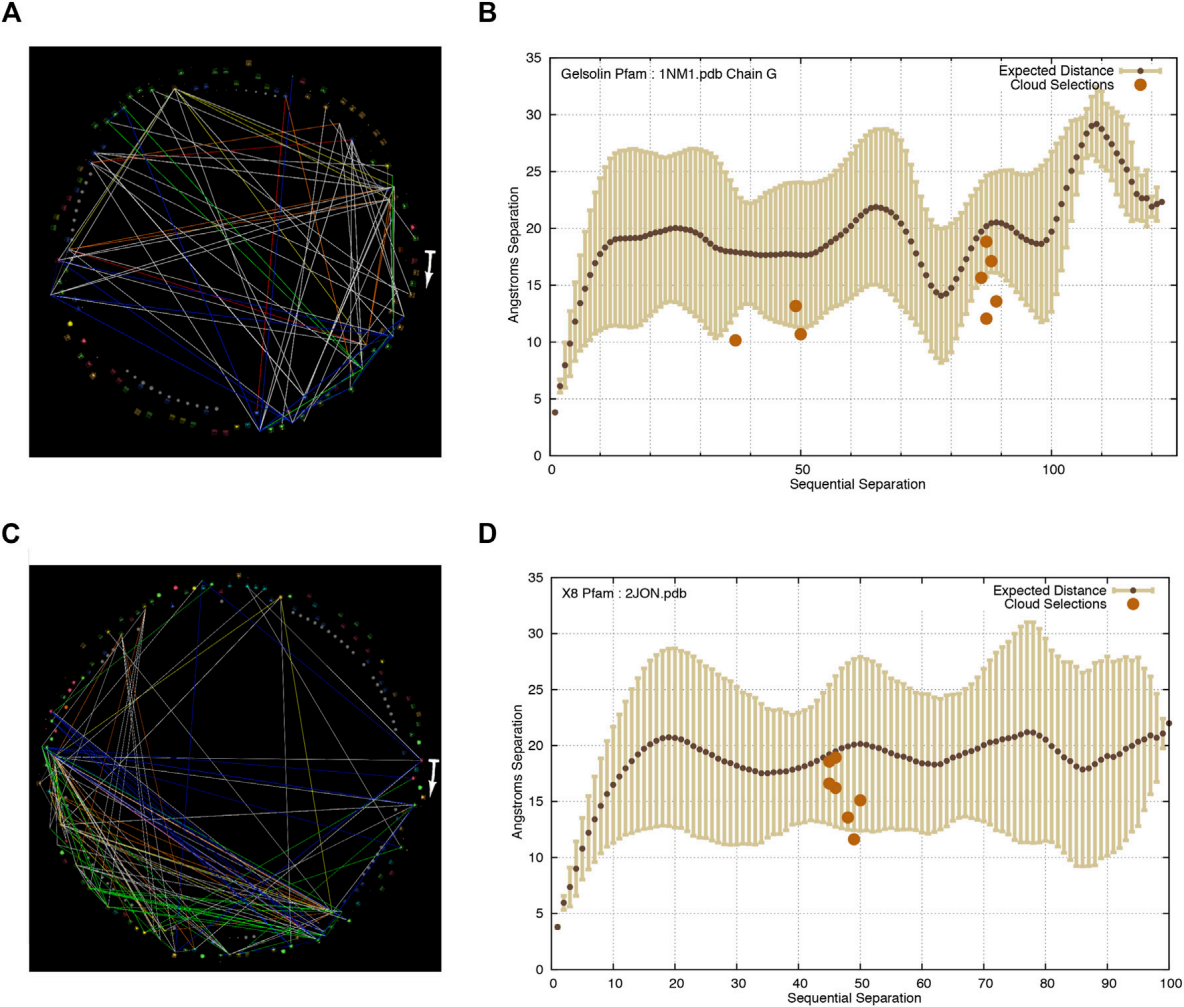
At similar thresholds of *T*_*r*_ and *P* as seen in ADK, other protein families display similar “clouds” of weakly co-evolving residue pairs, and similar areas of visually-random or node-link-free space. These residues linked by these weak co-evolution statistics are universally closer than the expected distance for residues of similar sequential separation in their protein structures. The start of the arrows in each image indicates the N-terminal of each protein and it points in the direction of increasing sequence coordinates. It should be noted that there are fewer distances plotted in the inter-residue distance plots than in the StickWRLD diagram of co-evolving residues, because the inter-residue distance plot shows distances for only those residue pairs that occur in each specific PDB file, while the StickWRLD diagram shows all co-evolution across each PFam family. **(A)** Correlated evolution statistics in the gelsolin domain family visualized as node-links in StickWRLD. *T*_*r*_ > = 0.12, *p* < = 0.05. **(B)** Residue pairs selected by a user as interesting in the StickWRLD diagram, plotted against the inter-residue distance distribution for the gelsolin Pfam family, with distances as found in Chain A of the 1NM1 PDB structure. **(C)** Correlated evolution statistics in the X8 family visualized as node-links in StickWRLD. *T*_*r*_ > = 0.1, *p* < = 0.001. **(D)** Residue pairs selected by a user as interesting in the StickWRLD diagram, plotted against the inter-residue distance distribution for the X8 Pfam family, with distances as found in the 2JON PDB structure.

In two out of three of the new Pfam protein families analyzed in this study (gelsolin and X8, results shown in Figure 2), as well as in an additional cloud detected in adenylate kinase, all but one of the residue pairs were closer than expected in the protein structures.

Ozer (2008) and Ozer and Ray (2007) provide an extensive analysis of the performance of *T*_*r*_ and significance across the entire Pfam and PDB databases for selecting close residue pairs. At the *T*_*r*_ and significance thresholds chosen (noted in the subfigure captions) the correlations displayed in these diagrams are only expected to be in contact approximately 20–30% more frequently than randomly-chosen residue pairs.

The residues selected in gelsolin chain G fall in the visually salient triangular and square patterns of interdependencies visible in Figure 2A. Those selected in X8 were in the dense triangular grouping in the lower left of the StickWRLD diagram shown in Figure 2C. The distances between the residues in each pair that occur in their relevant PDB structures are plotted in Figures 2B,D respectively. A single residue pair in the group selected in ADK fell above the expectation.

**FIGURE 3:**
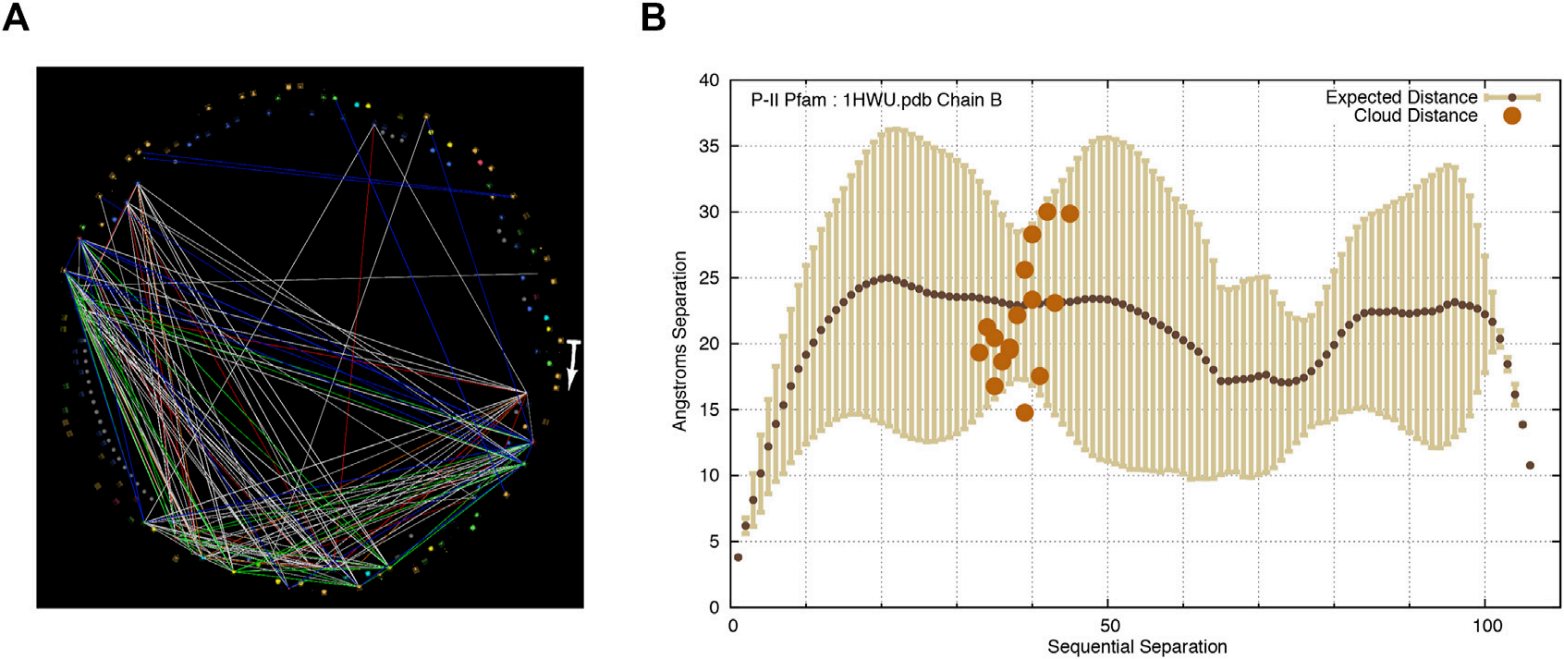
In P-II, an unmistakable cluster of co-evolving residues appears at similar *T*_*r*_ and *P* thresholds, but the distances between the implicated residues are scattered both above and below the expected distance for residues of similar sequential separation. The start of the arrow in subfigure **(A)** indicates the N-terminal of the protein and it points in the direction of increasing sequence coordinates. **(A)** Correlated evolution statistics in the P-II family visualized as node-links in StickWRLD. *T*_*r*_ > = 0.11, *p* < = 0.005. **(B)** Residue pairs selected by a user as interesting in the StickWRLD diagram, plotted against the inter-residue distance distribution for the P-II Pfam family, with distances as found in Chain B of the 1HWU PDB structure. The unexpectedly long inter-residue distances found for several of the cloud picks, appear to be due to the actual related pairs occurring in neighboring chains of the P-II multimer (Figure 4), rather than entirely within a single monomer subunit.

In the case of P-II we observed a different, seemingly incongruous result, as shown in Figure 3. In P-II, the user selected the residue pairs involved in the striking trapezoidal cluster shown in Figure 3A. Unlike with gelsolin and X8, as seen in Figure 3B, almost half of the selected residue pairs fall above the expected distance.

**FIGURE 4:**
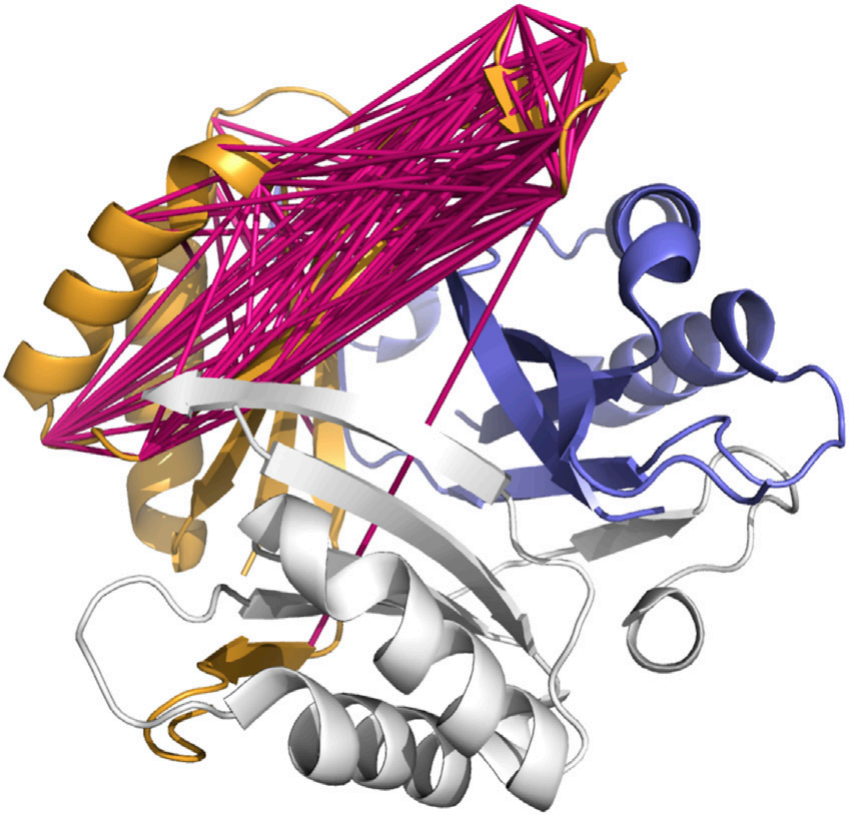
The structure of the homotrimeric functional P-II signal transduction protein, one unit of the trimer shown in orange, one in white, and one in blue. Links are shown in one subunit between the residues selected as co-evolving in the Pfam family PF00543 seed alignment. The blue helices at the upper right are the blue subunit’s copy of the orange helices to the trimeric structure’s left. The unexpectedly distant residue pairs seen in Figure 3 may be due to the co-evolution being between the blue subunit and orange subunit, rather than entirely within the orange (or any other single) subunit.

Examination of the protein structure for P-II (PDB ID 1HWU) however, suggests an explanation: Functional P-II is a homotrimer. Three identical copies of the protein assemble, as shown in Figure 4, into a near-symmetric triangular barrel (Machado Benelli et al., 2002).

Within a subunit (orange), the unexpectedly-long correlations span from the pair of short beta sheets at the top (12 to 1 o’clock), to regions of the protein at the far left (7 o’clock, 9 o’clock areas) of the image. However, the corresponding sequence regions of the blue subunit are at the upper right (2 o’clock).

Much as it is challenging for NMR experiments to differentiate between intramonomer and intermonomer contacts without additional data (O’Donoghue and Nilges, 2002; Sgourakis et al., 2011), simple co-evolution analysis cannot differentiate between co-evolution occurring within a single subunit or between neighboring subunits. It is, however, suggestive that the residues that co-evolve in the neighboring subunit are closer than the expected distance within a single subunit.

### Consistency of Visual Salience

When the cohort of 32 computer science (CS) students was presented with the static StickWRLD images shown here, their selections for “visually interesting” regions were essentially identical to those selected by the student of protein biophysics. There were small differences in the exact boundaries of their selections, and because we asked them to select regions instead of individual node-links their results are difficult to compare precisely with the results from our experienced student. However, qualitatively, the selections made by 30 out of 32 CS students were consistent with the results from the biophysics student. The two CS students whose results differed, each independently selected different empty regions of the figures because they felt that the absence of anything in those regions was the most interesting feature.

These results have been mimicked in audience-participation sessions in conference presentations to groups of artists, statisticians, and biologists.

When presented with the randomized StickWRLD diagrams (not shown), the cohort of CS students produced results with little consistency in terms of selected interdependencies. There were no consistent regions selected, though some selections overlapped. Several students did indicate one or more specific individual (always the brightest in the image) interdependencies as their selection. However, perhaps the most interesting result was that while none of the students showed the slightest hesitation to indicate that some portion of the real diagrams was interesting, most were reluctant to make any selection from the randomized diagrams, and several outright refused. One paper was marked “This is stupid”.

## Discussion

Our results suggest something interesting: while patterns of frequently co-evolving residues are implicated in protein function, it appears that patterns of infrequently co-evolving residues are implicated in protein structure. This makes evolutionary sense. Evolution favors systems that are robust to mutational insults. Therefore if a pair of residues is observed to co-evolve with great frequency, this is strong evidence for that pair of residues being required to maintain function (fitness). Structure however is under less evolutionary pressure than function, and so evolution may accept a variety of structurally-compensating mutations to correct for the destabilizing effects of mutation at a residue whose function is purely structural.

If a structural-residue mutation can be compensated for by several different mutations, at several different residues, none of those correlated mutations will occur with great frequency. As a result, none are likely to attain strong statistical significance. This is exactly what we see in our “interesting clouds”: infrequently-occurring groups of co-mutations that occur in proximity to each other, and which apparently contain structural information.

This observation has profound implications for fields such as protein engineering. Improving the activity and stability of engineered proteins has traditionally relied on building proteins that are “like what nature most-often chooses to do”. However, determining what information to include when engineering a protein has been challenging. Identifying the most frequently used residues, or consensus, from multiple sequence alignments (MSAs) has long been assumed to be the best way to determine the ideal residue composition of a protein. More recently it has been shown that proteins engineered using the consensus as a guide are sometimes less active than any of the wild-type proteins (Dagan et al., 2013; Risso et al., 2014). This deficit occurs because the consensus ignores co-dependencies between residues that are required for function or structural stability. Our results demonstrate that it is not sufficient to simply look at the top of the list of the most-frequently co-evolving residues to identify candidates for improving this engineering effort.

It is interesting to note that many of the residue pairs implicated in the observed “interesting clouds”, are not within the canonical 8 Å distance typically assumed to be the cutoff distance for residue contacts. This again has important implications for protein engineering efforts. Not only does it suggest a requirement to look beyond 8 Å when considering the impact of a design change, it also suggests that a larger range of weakly-stabilizing co-mutations are available in the engineer’s palette of design choices. Because a typical protein may be biased towards its native structure by as little as the energy of a few hydrogen bonds (∼5−15kcal/mol) (Pace, 1975), and increasing protein stability beyond its native state has been shown to negatively impact activity (Schreiber et al., 1994; Beadle and Shoichet, 2002; Dagan et al., 2013), the protein engineer must walk a narrow energetic path when introducing changes into a protein sequence. The energetic impact of interactions with residues within the 8 Å sphere of immediate contacts is large because of their direct interactions, and there are only a few of them available by which an engineer can modulate the effects of a mutation’s impact on stability. Widening the search window of candidates for compensating mutations, and including many with more subtle contributions to the stabilizing energy, can provide the engineer with additional design flexibility.

We have evidence in other research for the importance of this weakly co-evolving, more distant variety of interaction between protein residues. We have previously designed various mutants of Triosephosphate Isomerase (TIM) and assayed them for structure and function. A pure consensus version of the TIM (cTIM), which in fact included many of the most-strongly co-evolving residue pairs, was found to be poorly folded and nearly inactive. A modified version of cTIM (ccTIM) which restored additional more weakly-co-evolving residue pairs was stable and as active as wild-type TIM (Mohan, 2017). In a different experiment, a collaborator examined the pattern of co-evolution between the WW domain of dystrophin and its binding partner *β*-dystroglycan (Huang et al., 2000). Of the five residues in dystrophin that were selected as members of the interesting weakly co-evolving group between the proteins, two are members of the seven known inter-protein contacts (Huang et al., 2000), two others are sequentially adjacent to a crystallographic-contact residue, and the last is separated from a crystallographic contact by only one residue (Supplementary Material).

Taken together, these results suggest there is information in the patterns of weakly co-evolving residues that occur in proteins, even where the statistical significance of any individual co-evolution signal is so poor as to be discarded as beneath the noise floor.

Presented together in an appropriate explorable visualization interface, the human visual system is capable of integrating these individually insignificant contributions and identifying meaningful subsets of the co-evolution signals which display a relationship to protein structure and stability. These findings have both practical applications in improving the available design space for protein engineering, as well as utility in shedding light on the question of why strongly co-evolving residue pairs have disappointed expectations for predicting structural contacts.

### Caveats and Limitations

Our results present a variety of acknowledged limitations: While we found 49 closer-than-expected (out of *n* = 50 tested) co-evolving residue pairs, this result is derived from a total of four protein families. We do not know whether this generalizes to all proteins, or whether it is restricted to only proteins with specific characteristics such as size or a particular predominant secondary structure. In addition, in any given protein there are vastly more structural interactions than appear as “interesting clouds”, and we neither know whether additional interactions may become visible with different parameter choices, or if what can be seen is limited to specific types of structural interactions. We also have yet to define a concrete set of criteria by which “interesting clouds” could be algorithmically selected. Users anecdotally claim that they select such clouds based on a convolution of node-link weight, density, consistency of direction and consistency of end-points, but the specifics remain under investigation. Finally we do not claim that our algorithmic approach for identifying interactions (*P* and *T*_*r*_) is ideal. Better statistical or algorithmic approaches such as Machine Learning can almost certainly provide significantly better lists of residues that co-evolve. In this brief research report we argue only that our Visual Analytics approach provides interesting insight into the biological origin of different patterns of co-evolution, with potentially important implications for understanding and using such lists of co-evolving residues.

## Data Availability

The original contributions presented in the study are included in the article/Supplementary Material, further inquiries can be directed to the corresponding author.

## Footnotes

1
www.stickwrld.org.
