# Supplementary material for: The Importance of Weakly Co-Evolving Residue Networks in Proteins is Revealed by Visual Analytics
Source: Front Bioinform. 2022 Apr 5;2:836526. doi: 10.3389/fbinf.2022.836526 (PMC9580873; doi:10.3389/fbinf.2022.836526)
Supplement: Supplementary file 1 [file DataSheet1.PDF]

Supplementary Material

1 SUPPLEMENTARY DATA

Residues pairs selected as members of "interesting clouds" by a user with training in protein biophysics and experience with StickWRLD. Note that more residue pairs are selected here, than there are distances reported, because not all selected residue pairs existed in the PDB structures in which the distances were calculated.

1.1 Co-evolving residue pairs selected in the Adenylate Kinase Pfam (PF00406) seed alignment

(assumes zero-based indexing)

```
21 I 74 D
22 P 74 D
24 I 74 D
24 I 70 Q
21 I 67 R
22 P 67 R
24 I 67 R
30 F 67 R
36 K 67 R
33 A 62 G
30 F 60 T
36 K 64 V
22 P 61 I
25 A 64 I
24 I 64 V
```

1.2 Co-evolving residue pairs selected in Chain G of the Gelsolin family (PF00626) Pfam seed alignment

(assumes zero-based indexing)

```
08 L 97 Q
08 L 96 S
08 L 95 G
10 D 97 Q
10 D 96 S
08 L 51 R
08 L 50 W
08 L 97 I
10 D 51 R
10 D 50 W
10 D 47 I
51 R 96 S
```

```
51 R 97 Q
50 W 96 S
50 W 97 Q
47 I 96 S
47 I 97 Q
```

1.3 Co-evolving residue pairs selected in the P-II family Pfam (PF00543) seed alignment

(assumes zero-based indexing)

```
38 R 71 E
38 R 72 Y
38 K 73 Y
38 R 74 V
38 R 77 F
34 K 71 E
34 K 72 Y
34 K 74 V
34 K 77 F
32 E 71 E
32 E 72 Y
32 E 74 V
32 E 77 F
36 F 71 E
36 F 77 F
36 R 73 Y
```

1.4 Co-evolving residue pairs selected in the X8 domain family Pfam (PF07983) seed alignment

(assumes zero-based indexing)

```
27 A 72 A
27 A 73 F
24 F 69 L
23 L 69 L
24 L 72 A
24 L 73 F
23 A 73 F
```

1.5 Co-evolving residue pairs selected between the Dystrophin WW domain and β-dystroglycan

(dystrophin to β-dystroglycan)

```
3064 K 144 V
3072 Y 117 P
3074 V 144 V
3074 V 162 R
```

```
3077 N 153 D
3077 N 178 T
3080 T 132 A
3080 T 142 Q
3080 T 144 V
```
